# The overexpression of p16 is not a surrogate marker for high-risk human papilloma virus genotypes and predicts clinical outcomes for vulvar cancer

**DOI:** 10.1186/s12885-016-2503-y

**Published:** 2016-07-13

**Authors:** Jacek J. Sznurkowski, Anton Żawrocki, Wojciech Biernat

**Affiliations:** Department of Surgical Oncology, The Medical University of Gdańsk, ul. Smoluchowskiego 17, 80-214 Gdańsk, Poland; Department of Pathology, The Medical University of Gdańsk, ul. Smoluchowskiego 17, 80-214 Gdańsk, Poland

**Keywords:** Vulvar cancer, vSCC, HPV, p16, Prognosis

## Abstract

**Background:**

We aimed to evaluate the correlation between p16^ink4a^-overexpression and high risk (hr)HPV-DNA in vulvar squamous cell carcinoma (vSCC) tumors as well as the impact of both biomarkers on the prognosis of vSCC patients.

**Methods:**

PCR-detection of (hr)HPV-DNA and immunohistochemical staining for p16^ink4a^ were conducted in 85 vSCC tumors. Survival analyses included the Kaplan–Meier method, log-rank test and Cox proportional hazards model.

**Results:**

p16^ink4a^-overexpression and (hr)HPV-DNA were detected in 35 and 37 of the 85 tumors, respectively. Among the 35 p16^ink4a^-positive tumors, 10 lacked (hr)HPV-DNA (29 %). Among the 50 p16^ink4a^-negative tumors, (hr)HPV-DNA was detected in 12 cases (24 %). The median follow-up was 89.20 months (range 1.7–189.5 months). P16^ink4a^-overexpression, but not (hr)HPV-DNA positivity of the primary tumor, was correlated with prolonged overall survival (OS) (*p* = 0.009). P16^ink4a^-overexpression predicted a better response to radiotherapy (*p* < 0.001). Univariate analysis has demonstrated that age (*p* = 0.025), tumor grade (*p* = 0.001), lymph node metastasis (*p* < 0.001), FIGO stage (*p* < 0.001), p16^ink4a^-overexpression (*p* = 0.022), and adjuvant RTX (*p* < 0.001) were prognostic factors for OS. Multivariate analysis has demonstrated that lymph node metastasis (HR 1–2.74, 95 % CI 1.50–5.02, *p* = 0.019), tumor grade (HR 1–2.80, 95 % CI 1.33–5.90, *p* = 0.007) and p16^ink4a^-overexpression (HR 1–2.11, 95 % CI 1.13–3.95, *p* = 0.001) are independent prognostic factors.

**Conclusion:**

The discovered overlap suggests the use of p16^ink4a^ in combination with HPV-DNA detection as an ancillary test for future research and clinical studies in vSCC. The prognostic and predictive value of p16^ink4a^-overexpression should be tested in larger cohort studies.

**Electronic supplementary material:**

The online version of this article (doi:10.1186/s12885-016-2503-y) contains supplementary material, which is available to authorized users.

## Background

Vulvar cancer has an incidence of 1–2 per 100,000 women per year and represents 3–5 % of all gynecological malignancies. The most common type of vulvar cancer is vulvar squamous cell carcinoma (vSCC) [1]. Two different etiopathogenic pathways have been shown to be involved in vSCC development: one is induced by transforming infections in human papillomavirus (HPV) high-risk (hr) genotypes, and the other arises in the absence of HPV in the setting of long-standing dermatosis [2]. Histologically, HPV-positive vSCCs are of the basaloid or warty type and arise from the vulvar intraepithelial neoplasia (VIN) of the usual type. In HPV-transformed cells, the downstream p16^ink4a^-CDK4-pRB pathway is blocked by the inactivation of pRB through the HPV E7 protein, and it results in the nuclear and cellular accumulation of the cyclin-dependent kinase inhibitor p16^ink4a^ [2, 3]. Although it was suggested that p16^ink4a^-overexpression in vulvar cancer correlates with the presence of HPV [4], a recent study revealed substantial mismatch between p16^ink4a^-overexpression and HPV status [5].

HPV-dependent and HPV-independent vSCCs were suggested to be separate entities [2]; however, the prognostic impact of the HPV etiology in vSCC is controversial [6].

The aim of this study was to assess the prevalence of HPV genotypes and the concordance between the presence of (hr)HPV-DNA and p16^ink4a^overexpression within vSCC tumors. The secondary aim was to analyze the prognostic significance of p16^ink4a^ and (hr)HPV-DNA status in vSCC patients.

## Methods

This retrospective study was approved by the Polish Ministry of Science and Higher Education review board (decision number for approval 2835/B/P01/2009/36). The board determined that further informed consent was not required, as all patients provided informed consent for tissue sampling prior to surgical treatment, including written consent for the storage of their information in the hospital database and the use of their information for research.

### Patients and specimens

We studied 85 patients with primary vSCC who underwent surgical treatment at the Department of Gynaecological Oncology at The Medical University of Gdańsk between January 2002 and December 2007. All patients underwent standard surgical treatment, which was not modified by the results of the sentinel node procedure. A wide local excision was performed if the tumor diameter did not exceed 2 cm and the depth of invasion was less than 1 mm. In cases of lateral tumors with an invasion greater than 1 mm, a wide local excision or tailored radical vulvectomy with a bilateral inguinofemoral lymphadenectomy was performed. Lymphadenectomies were mostly performed with separate incisions. Postoperative radiotherapy was administered to all patients with positive inguinal lymph nodes, with the exception of those patients who had both a well-differentiated histology of the primary tumor and also only one lymph node metastasis. Overall, 33 (39 %) patients received adjuvant radiotherapy. Clinical data were obtained from the medical records and questionnaires designed specifically for our previous studies conducted in the same cohort [7, 8].

Histopathological data on 76 tumors were obtained from two previous studies [7, 8]. Nine new tissue specimens were added (collected from consecutive patients treated in 2007) and newly reviewed with the same pathological criteria.

Tumor type (pT), depth of invasion (measured from the epithelial-dermal junction of the adjacent, most superficial dermal papillae to the deepest point of invasion), tumor grade according to the GOG (Gynecological Oncology Group) and lymph nodes status (pN) were verified by the same two independent pathologists (without knowledge of the disease outcome). All of these patients were staged according to the new 2009 FIGO system for vulvar cancer [9].

Finally, immunohistochemical (IHC) staining for p16^ink4a^ and PCR detection of HPV-DNA were performed on 85 paraffin-embedded tissue samples from the primary tumors.

#### Antibodies

Mouse anti-human p16^ink4a^ monoclonal (sc-56330) antibody was obtained from Santa Cruz Biotechnology (USA).

#### Immunohistochemistry

For immunohistochemical staining, four-micron-thick serial sections were cut, placed onto slides, and deparaffinized. For epitope retrieval, slides were immersed in Target Retrieval Solution (pH 6.0; Dako Cytomation, Denmark) and heated in a pressure cooker. The slides were incubated for 90 min with primary antibodies. The reaction was visualized using the Novolink Polymer Detection System (Novocastra Laboratories). Appropriate positive and negative controls were included for each case. As a positive control, a case of HPV-related cervical cancer was used. For the negative control, the primary antibody was replaced with normal mouse IgG at an appropriate dilution. Immunohistochemistry results were evaluated by two independent pathologists, who were blind to the clinical data. The concordance rate between their observations was over 96 %.

### Evaluation and classification of p16^ink4a^ immunostaining

The evaluation of the p16^ink4a^ immunostaining was performed on 3 different staining patterns: negative, focal, and diffuse staining. For statistical purposes, p16 immunostaining was classified as positive or negative. Staining for p16^ink4a^ was considered positive only in cases with a strong diffuse and continuous nuclear/cytoplasmic expression of p16^ink4a^ within the cancer nests (focal and weak diffuse staining were considered negative).

### Detection of DNA HPV

#### Tissue dissection and DNA preparation

Genomic DNA was prepared from two to three 4 mm sections from each case using standard methods. DNA was obtained from the samples by incubating them with 250 μl proteinase K solution (1 mg/ml) for 16 h at 70 °C. Following heat inactivation at 95 °C for 10 min, 10 μl of the supernatant was used for PCR. Appropriate positive and negative controls were incorporated during the DNA preparation and subsequent testing to monitor test performance.

Specimen extracts were also tested by real-time PCR for the human RNase P gene to monitor specimen quality.

#### Mucosal HPV DNA amplification and genotyping

Broad-spectrum HPV DNA amplification and mucosal HPV genotyping was performed using the SPF10–LiPA25 system (SPF10 HPV LiPA, version 1; manufactured by Labo Biomedical Products, Rijswijk, The Netherlands), as described previously [10, 11]. All testing was commercially done in the DDL Diagnostic Laboratory in Rijswijk, The Netherlands, according to the instructions of the manufacturer. First, SPF10 PCR was used to amplify a 65-base pair fragment from the L1 region of the HPV genome. The amplimers from all samples were subsequently tested with the DNA Enzyme Immuno-Assay (DEIA). This method provides an optical density value and is able to detect the SPF10 amplimer from more than 68 HPV types. Amplimers from positive samples can be used to identify 25 individual HPV genotypes (high-risk HPV: 16, 18, 31, 33, 35, 39, 45, 51, 52, 56, 58, 59, 66, 68, 70, and low-risk HPV: 6, 11, 34, 40, 42–44, 53, 54, 74) simultaneously in a reverse hybridization assay (RHA) by hybridization to DNA probes attached to nitrocellulose strips. After the RHA, the strips were dried, and the purple colored bands were visually scored and interpreted by aligning them with the standard grid.

No aberrant results were observed in the sectioning, DNA isolation or PCR controls.

### Follow up analysis

The impact of the following variables on overall survival was assessed: type of the tumor (pT), lymph node status (pN), tumor grade, depth of invasion, FIGO stage, age and recurrence, as well as p16^ink4a^ and HPV-status.

### Statistical analysis

To determine statistically significant differences between the variables, the Mann-Whitney U test was used. Overall survival (OS) curves were estimated using the Kaplan-Meier method and compared using a two-sided log-rank test. Independent variables were first analyzed with univariate analysis. Variables shown by univariate analysis to be significantly associated with survival were entered into a Cox proportional hazards regression model for multivariate analysis. A *p* value <0.05 was considered significant. All analyses were performed using Statistica 10 software (Stat Soft Inc. USA).

## Results

### Study population

The clinicopathological features of the patients with primary vSCC and their relationships to the course of the disease are summarized in Additional file 1: Table S1 and Additional file 2: Table S2, respectively. The median age of the patients was 68 years (range 36–85 years), and the median duration of the overall follow-up was 89.20 months (range 1.7–189.5 months). The 5-year disease free survival (DFS) rate was 61.75 %. Recurrence was observed in 16 patients (16/85, 18.82 %); 13 had local recurrence (13/85, 15.29 %) and three revealed recurrence in the groin (3/85, 3.53 %).

The depth of invasion in pN-positive (median 8.2 mm) and pN-negative cases (median 6.0 mm) was significantly different (U–MW test, *p* = 0.000743).

### Immunohistochemical expression of p16^ink4a^

**Fig. 1:**
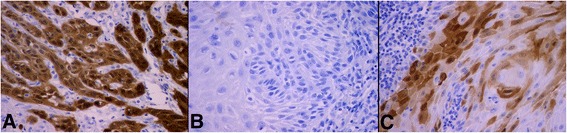
Microphotographs of immunohistochemical staining for p16^ink4a^: **a**. strong diffuse nuclear/cytoplasmic expression - p16^ink4a^ positive cases; **b**. p p16^ink4a^ negative cases; **c**. focal check-board pattern of expression - p16^ink4a^ -negative cases

Staining for p16 was performed within the nuclei and the cytoplasm of the cancer cells (Fig. 1a). The 85 patients were divided into 2 groups based on p16 status: p16^ink4a^-negative (*n* = 50) (Fig 1b, c) and p16^ink4a^-positive (*n* = 35) (Fig. 1a).

### Detection of (hr)HPV-DNA

HPV-DNA was detected in 38 of the 85 primary vSCC tumors (45 %).

The HPV genotype distribution is provided in Additional file 3: Table S3. HPV16 was the most prevalent type detected (33/38 cases, 86.8 % of infected tumors). HPV33 was found in 2 tumors, HPV56 in one tumor, and the simultaneous occurrence of 16 and 18, and 39 and 59 HPV types was identified in one tumor each. The low risk HPV6 genotype was detected in one tumor, and this case was indexed as (hr)HPV-DNA-negative. Finally, 85 patients were divided into 2 groups based on (hr)HPV-DNA status: negative (*n* = 48) and positive (*n* = 37).

### Overlap between p16^ink4a^-overexpression and (hr)HPV status

Among the 35 vSCC tissue samples with p16^ink4a^-overexpression, 10 cases lacked (hr)HPV-DNA (28.6 %). Among the 50 tumors without p16-overexpression, (hr)HPV-DNA was detected in 12 cases (24.0 %). The combined presence of (hr)HPV-DNA and p16-overexpression was detected in 25 of the 85 cases (29.4 %). P16^ink4a^ status in 85 vSCC cases in relation to (HR)HPV genotypes is summarized in Table 1.

**Table 1:** (HR)HPV genotype distribution and p16 status in 85 vSCC cases. (All samples were sufficient for HPV DNA testing)

*(HR)HPV-DNA*	Total number	P16-positive	P16-negative
negative	48	10	38
positive	37	25	12
type16	31	21	10
type 16 and 18	1	0	1
type 33	3	2	1
type 56	1	1	0
type 39 and 52	1	1	0

### Prognostic significance of p16^ink4a^

**Fig. 2:**
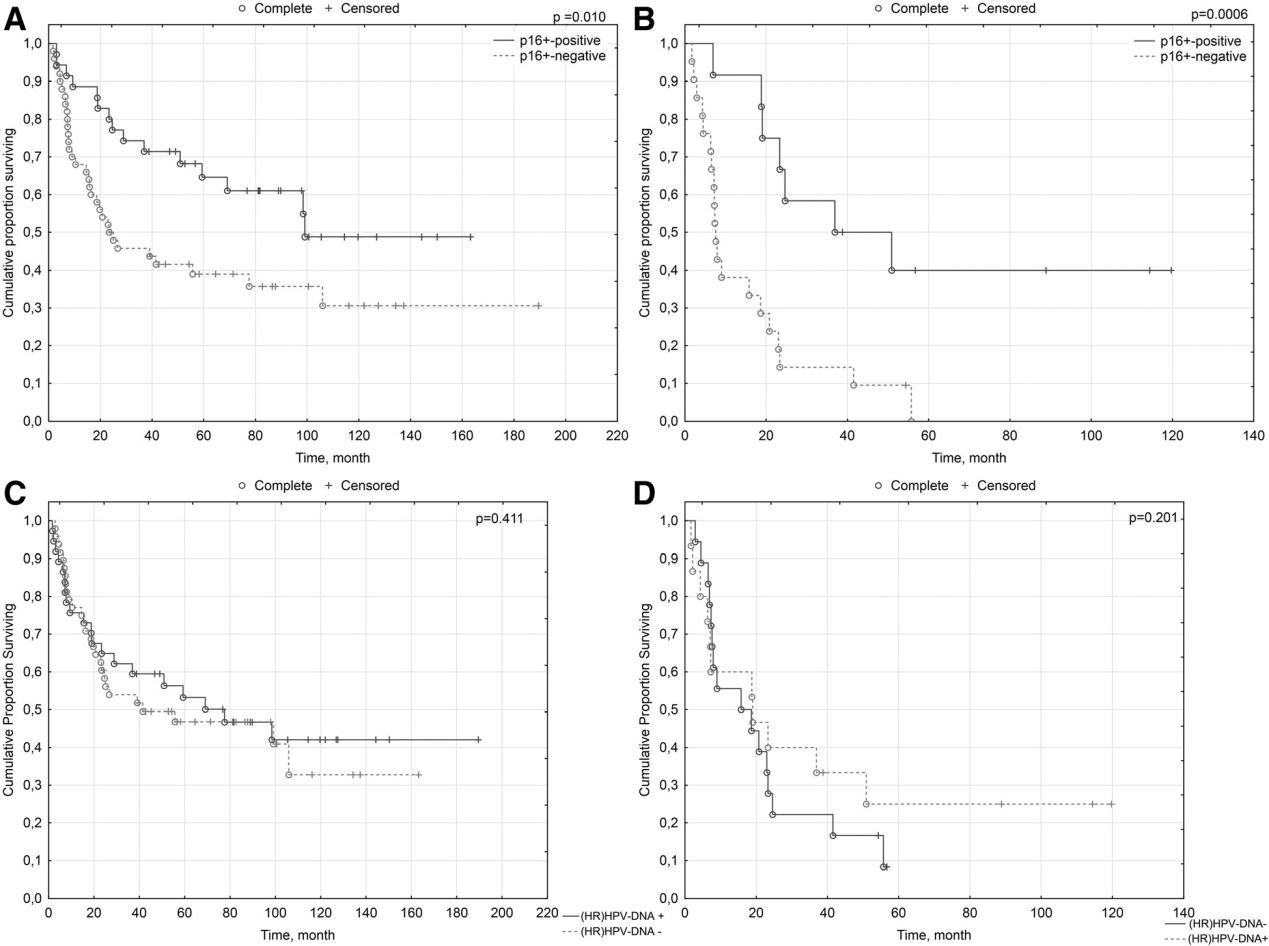
Prognostic significance of p16^ink4a^-overexpression: **a**. general patient cohort; **b**. patients requiring adjuvant radiotherapy; (hr)HPV-DNA: **c**. general patient cohort; **d**. patients requiring adjuvant radiotherapy

The OS (months) of patients with p16^ink4a^-positive and p16^ink4a^-negative vSCC was 98.97 and 23.33, respectively (*p* = 0.009). A positivity for p16^ink4a^ in the primary tumor correlated with a prolonged survival of vSCC patients (*p* = 0.010) (Fig. 2a). There was no significant difference in the proportion of p16^ink4a^-positive and p16^ink4a^-negative patients who received adjuvant radiotherapy (12/35 [34 %] vs. 21/50 [42 %]; *p* = 0.506). The median OS (months) of radiated patients with p16^ink4a^-positive and p16^ink4a^-negative vSCC was 36.93 and 7.47, respectively. A positivity for p16^ink4a^ predicted longer OS among vSCC patients who received adjuvant radiotherapy (*p* = 0.0006) (Fig. 2b).

### Prognostic significance of (hr)HPV-DNA

The median OS (months) of patients with (hr)HPV-DNA-positive and (hr)HPV-DNA-negative vSCC was 69.31 and 41.02, respectively (*p* = 0.41). A positivity for DNA of (hr) HPV in the primary tumor was not correlated with a prolonged survival of vSCC patients (*p* = 0.411) (Fig. 2c). There was no difference in the proportion of (hr)HPV-DNA-positive and (hr)HPV-DNA-negative patients who received adjuvant radiotherapy (15/37 [41 %] vs. 18/48 [37 %]; *p* = 0.825). The OS (in months) of radiated vSCC patients with tumors positive and negative for (hr)HPV-DNA was 18.92 and 15.77, respectively. A positivity for (hr)HPV-DNA did not predict longer OS among vSCC patients who received adjuvant radiotherapy (*p* = 0.201) (Fig. 2d).

### Prognostic value of other clinicopathological variables

#### Recurrence

**Fig. 3:**
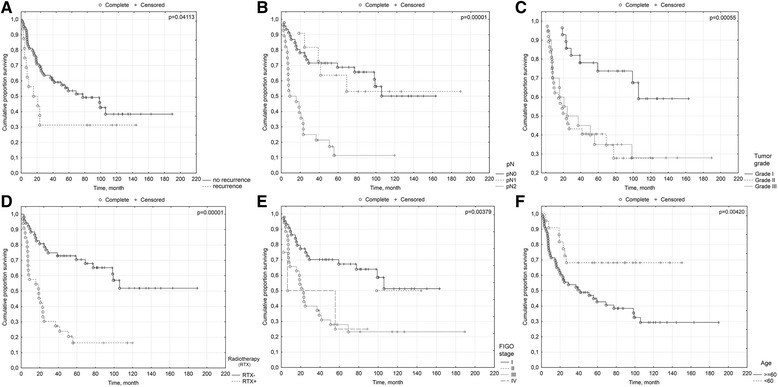
Prognostic significance of clinicopathological variables: **a**. recurrence: no reccurence/recurrence; **b**. pN: pN0/pN1/pN2; **c**. Tumor grade: G1/G2/G3; **d**. Adjuvant radiotherapy: RTX-/RTX+; **e**. FIGO stage: I/II/III/IV; **f**. Age: > = 60/<60 years

Recurrence was correlated with decreased overall survival (*p* = 0.0411) (Fig. 3a).

#### pT and pN status (according to the TNM system)

Most of the cases were staged as T1 (76/85, 89.41 %). Tumor type (pT: T1 (*n* = 76), T2 (*n* = 8), T3 (*n* = 1), T4 (*n* = 0)) has not revealed any influence on overall survival (*p* = 0.5027).

Nodal status (pN: N0 vs. N1/N2) has a significant impact on overall survival (*p* = 0.00006) (Fig. 3b).

#### Histological tumor grade

We found significant differences in the overall survival between patients with different histological tumor grades (divided in accordance with the three-tier grading scheme: G1/G2/G3) (*p* = 0.00055) (Fig. 3c), as well as between cases with well-differentiated (differentiation grade 1) and poorly-differentiated tumors (differentiation grades II–III) (*p* = 0.00021).

#### Depth of invasion

We did not manage to find any borderline depth of invasion with a significant impact on overall survival (for 7 mm (median) *p* = 0.057).

#### Adjuvant radiotherapy

Thirty-three patients who received adjuvant radiotherapy had a significantly worse prognosis compared to patients (*n* = 52) without irradiation (*p* = 0.00001) (Fig. 3d).

#### FIGO stage

The stage distribution according to the 2009 FIGO staging system was as follows: stage I: 44 (51.76 %), stage II: 2 (2.35 %), stage III: 35 (41,18 %) and stage IV 4 (4,71 %). The cumulative 5-year survival was as follows: stage I: 67 %, stage II: no data (too small of a group- 2 cases), stage III: 27 % and stage IV: 25 % (*p* = 0.00379) (Fig. 3e).

#### Age

Patients older than 60 years had a significantly worse prognosis (*p* = 0.004) (Fig. 3f).

### Univariate and multivariate analyses of prognostic variables in vSCC patients

In the univariate analysis, age (*p* = 0.0254), lymph node status (*p* = 0.0005), tumor grade (G1 vs. G2 + G3) (*p* = 0.001), adjuvant radiotherapy (*p* = 0.000005) and p16-overexpression (*p* = 0.0216) were prognostic factors for OS (Table 2). Multivariate analysis revealed that p16-expression (hazard ratio [HR] = 2.11, 95 % confidence interval [CI] = 1.13–3.95, *p* = 0.001), nodal status (HR = 2.74, 95 % CI = 1.50–5.02, *p* = 0.019), and tumor grade (HR = 2.80, 95 % CI = 1.33–5.90, *p* = 0.007) were independent prognostic factors for OS (Table 3).

**Table 2:** Univariate analyses of survival in vulvar cancer patients

Variables	Categories	Overall survival		p
		HR	95 % CI	
Nodal status	Negative for metastases	1	1.59–5.22	0.0005
	Positive for metastases	2.88		
Adjuvant RTX	Yes	1	2.22–7.30	0.000005
	No	4.02		
Histologic grade	Low (G1)	1	1.65–7.15	0.001
	High (G2 + G3)	3.43		
p16 status	Positive	1	1.11–3.81	0.0216
	Negative	2.06		
FIGO stage	I, II, III, IV	1.62	1.23–2.12	0.000592
Depth of invasion	Continuous	1.05	0.96–1.15	0.283261
Age	<60	1	1.12–5.59	0.025436
	>60	2.5

**Table 3:** Multivariate analyses of survival in vulvar cancer patients

Variables	Categories	Overall survival		p
		HR	95 % CI	
Nodal status	Negative for metastases	1	1.50–5.02	0.019
	Positive for metastases	2.74		
Histologic Grade	Low (G1)	1	1.33–5.90	0.007
	High (G2 + G3)	2.80		
p16 status	Positive	1	1.13–3.95	0.001
	Negative	2.11		

## Discussion

The prevalence of HPV-positive tumors in our series was nearly 44.7 %. Previous studies on vSCC have reported highly variable numbers of HPV-positive cases that have ranged between 0 and 66 % depending on the method of HPV detection and the histological types of vSCC analyzed [12–15].

In 2013, de Sanjosé S et al. reported 28.6 % HPV-positive cases among 1709 invasive vulvar cancers (IVC) [5]. An overestimation of HPV positivity in our cohort could be generally explained by different case selection, as both studies were conducted in the same Laboratory (DDL Diagnostic Laboratory in Rijswijk, The Netherlands) with an identical protocol using SPF-10 broad-spectrum primers and genotyping with a reverse hybridization line probe assay (LiPA25).

Indeed, all of our cases come from Poland (Europe) while only 49.8 % of women with IVC included in de Sanjosé’s study were European. The prevalence of HPV-DNA was found to be higher among European women than women living in other geographical regions [5].

Additionally, the number of well differentiated tumors was higher in our group than in IVC cohort described by Sanjosé S et al. (76 % vs. 71 %).

Indeed, the proportion of histological types is crucial for cohort prevalence, as it was shown that HPV-positivity among warty-basaloid and keratinizing vSCC tumors varies, and it was described as 69 and 11.5 % of cases, respectively [5].

Our study confirmed the predominant contribution of HPV-16 to the etiology of HPV-related vulvar cancer and suggested that other HPV types, such as HPV-33, HPV-18 and HPV-56, which are common in cervical cancer, are also important to vulvar carcinogenesis, although to a lesser degree.

In 28.6 % of p16^ink4a^-positive tumors, a lack of (hr)HPV-DNA was observed, and in 24.0 % of p16^ink4a^-negative tumors, (hr)HPV-DNA was detected. The substantial mismatch between p16^ink4a^-overexpression and HPV-status reported here was also observed in the largest cohort of 1709 vSCC cases [5]. Seventeen percent of tumors expressing p16^ink4a^ lack (hr)HPV-DNA, and 9.4 % of tumors lack p16^ink4a^-overexpression despite the presence of (hr)HPV-DNA [5].

A lack of (hr)HPV-DNA in p16-positive tumors could be explained by the fact that the HPV oncoprotein, E7, which functionally inactivates RB, is not the only thing responsible for the increases in p16^ink4a^ expression [16]. Although it is believed that RB inactivation is a requisite for the elevation of p16^ink4a^ expression in cancer [2, 3], aberrations in the RB pathway are not obvious in every tumor. The RB checkpoint is deregulated by multiple mechanisms independent of *RB1* mutation, deletion or methylation. The viral oncogene expression represents just one potential form of multiple possible ways of RB inactivation [16].

Several findings have proven the strong association between age-promoting, ‘gerontogenic’ signals and p16^ink4^ expression [16]. Thus, the impact of senescence and inflammation on p16^ink4^ expression in our older age cohort of vSCC patients should also be considered.

There remains the possibility that a certain fraction of HPV-negative samples were false negatives. However, during the testing, we checked all the samples for amplifiable human genomic DNA. All samples showed the presence of human DNA by PCR (RNAseP gene). The size of the PCR fragment in this test is also 65 base pairs, and therefore, it is most sensitive PCR for formalin-fixed paraffin-embedded/FFPE/tissue samples.

(hr)HPV-DNA-positive cancer cases without marked p16-overexpression could be explained by the fact that close to half of all human cancers show p16^Ink4a^-inactivation, ranging from 25 to 70 % [17]. Such an event could exist parallel to functional inactivation of RB by the E7 protein. Some of the HPV-positive samples could also be false positive. By performing Laser Capture Microdissection [18], it is possible to assign HPV types to the lesional cells themselves; however, it was not performed in the current study. Therefore, we cannot exclude the possibility of contamination of the cancer samples by HPV virions from the surrounding vulvar epithelium.

Taking these facts together, we postulate not to treat p16^ink4a^-overexpression as a surrogate marker for (hr)HPV infection in vSCC. The correlation between p16^ink4a^ and (hr)HPV-DNA varies in squamous cell carcinomas. In cervical cancer, p16^ink4a^ overexpression and (hr)HPV status are quite well correlated [19], while in oral cancer, a lack of concordance is frequently reported [20].

The combined presence of (hr)HPV-DNA and p16^ink4a^-overexpression was detected in 25 of the 85 cases (29.4 %). This result is in the range of the series reported by de Sanjosé S et al., who have reported 22.4 % HPV-driven cases out of 1709 vSCCs [5]. Probably, this is the real contribution of the HPV infection to vSCC development.

In the following analyses, we assessed the prognostic significance of (hr)HPV-DNA status and p16 overexpression separately. The (hr)HPV-DNA status of the primary tumor has no impact on the survival of vSCC patients. P16-overexpression was found to be prognostic, and also predicted a better response to radiochemotherapy.

Several reports investigating the relationship between HPV DNA and vSCC prognosis have produced conflicting results [6, 21–24]. Two old studies from the early 1990s [23, 24] reported a better survival in DNA HPV-positive patients, but their results are both hampered by the limited number of cases included (55 and 60, respectively) and the tests used for HPV detection. In recent years, one paper (with a median follow up of 42 months) confirmed a prolonged survival in patients with vSCC tumors positive for high risk DNA HPV [22], but two others (with a longer follow up) denied the prognostic significance of HPV DNA within cancer tissue [6, 21].

We identified only two studies that utilized p16 expression for the survival analysis of vSCC patients, and they reported contradictory results [6, 25]. Our results were consistent with the findings of Tringler et al. [25], but they were in opposition to the results of Alonso et al. [6], who did not identify p16 status as a prognostic indicator of vSCC. The low prevalence of p16-positive tumors (19 % [19/98]) might explain the lack of prognostic significance of p16 status in the Alonso et al. study, whereas the percentage of p16-positive cases in our study and that by Tringler et al. [25] was 41 % (35/85) and 43 % (34/80), respectively. The median follow-up in the Alonso et al. cohort was only 45 months [6], while it was 52 and 89 months in the Tringler study [25] and our study, respectively.

The conducted univariate and multivariate analyses revealed that p16-overexpression is an independent prognostic factor with respect to survival.

RB inactivation releases p16^Ink4a^ from its negative feedback control, causing a paradoxical increase in the levels of this protein, which attempts to inhibit uncontrolled cellular replication [26]. Thus, it is not surprising that p16^ink4a^-overexpression itself (not the HPV virus) has a protective role in HPV-related malignancies.

It was shown that cancers that present p16^Ink4a^-overexpression are very sensitive to radiotherapy, and they have a better prognosis [27]. A better response to radiochemotherapy has been associated with a more favorable prognosis of HPV-positive head and neck cancers [28–32]. Indeed, we also confirmed that p16^Ink4a^-overexpression predicts a better clinical outcome among patients requiring adjuvant radiotherapy. The two compared groups (irradiated p16-positive and p16-negative) were similar in terms of the number of positive nodes and the presence of extracapsular spread, which strongly supports this conclusion.

This study has the traditional weaknesses of a retrospective design, and the results obviously represent a small cohort. Lack of information on smoking and cause of death potentially limit the prognostic analysis. The strengths of the study include the treatment of patients according to uniform standards and a sufficient follow-up duration to reveal recurrences and to allow for the reliable assessment of the prognostic significance of all analyzed biomarkers. Data on (hr)HPV-DNA prevalence were provided by highly experienced sources in the HPV-DNA detection laboratory.

## Conclusions

The overexpression of p16^ink4a^ is not a surrogate marker for a transforming infection with HPV high-risk genotypes in vSCC. This suggests the use of p16^ink4a^ in combination with HPV DNA-detection as an ancillary test for research and clinical studies when HPV is not a necessary cause. The prognostic and predictive significance of p16^Ink4a^-overexpression within cancer tissue requires further investigation in future prospective studies.

## Abbreviations

DFS, disease free survival; DNA, deoxyribonucleic acid; FIGO, fr. Fédération internationale de gynécologie et d’obstétrique; GOG, gynecologic oncology group; HPV, human papilloma virus; HR, hazard ratio; hr, high risk; IHC, immunohistochemistry; IVC, invasive vulvar cancer; OS, overall survival; p16^Ink4a^, protein, cyclin-dependent kinase inhibitor 2A, multiple tumor suppressor 1; PCR, polymerase chain reaction; vSCC, vulvar squamous cell carcinoma

## Additional files

Additional file 1: Table S1. Clinicopathological characteristic of the vSCC patients. (DOCX 13 kb) Additional file 2: Table S2. Clinical and histopathological characteristics of the vSCC patients related to the course of the disease. (DOCX 13 kb) Additional file 3: Table S3. HPV genotype distribution and p16 status in 85 vSCC cases. This file contains the dataset supporting the conclusions. (DOCX 13 kb)
