# Supplementary material for: The overexpression of p16 is not a surrogate marker for high-risk human papilloma virus genotypes and predicts clinical outcomes for vulvar cancer
Source: BMC Cancer. 2016 Jul 13;16:465. doi: 10.1186/s12885-016-2503-y (PMC4944532; doi:10.1186/s12885-016-2503-y)
Supplement: Additional file 1: Table S1. — Clinicopathological characteristic of the vSCC patients. (DOCX 13 kb) [file 12885_2016_2503_MOESM1_ESM.docx]

Supplementary Table 1. Clinicopathological characteristic of the vSCC patients

| vulvar SCC patients (n=85) median follow up 89,20 months (range 1.7-189.5) | | |
| --- | --- | --- |
| Age, /median/ | 68 (range 36-85) years | |
| depth of invasion, /median/ | 7.03 (range 0.5-18) mm | |
| Grading  G1/G2+G3 | G1 | 28 (32.9%) |
|  | G2+G3 | 57 (67.1%) |
| Grading  G1/G2/G3 | G1 | 28 (32.9%) |
|  | G2 | 37 (43.5%) |
|  | G3 | 20 (23.6%) |
| pT | pT1a | 1 (1.2%) |
|  | pT1b | 22 (25.9%) |
|  | pT2 | 56 (65.8%) |
|  | pT3 | 5 (5.9%) |
|  | pT4 | 1 (1.2%) |
| pN | pN0 | 30 (35.3%) |
|  | pN1 | 13 (15.3%) |
|  | pN2 | 26 (30.6%) |
|  | pNx | 16 (18.8%) |
| FIGO stage | I | 44 (51.8%) |
|  | II | 2 (2.4%) |
|  | IIIa | 8 (9.4%) |
|  | IIIb | 20 (23.5%) |
|  | IIIc | 7 (8.2%) |
|  | IVA | 4 (4.7%) |
| Recurrence | yes | 16 (18.8%) |
|  | no | 69 (81.2%) |
