# Supplementary material for: The overexpression of p16 is not a surrogate marker for high-risk human papilloma virus genotypes and predicts clinical outcomes for vulvar cancer
Source: BMC Cancer. 2016 Jul 13;16:465. doi: 10.1186/s12885-016-2503-y (PMC4944532; doi:10.1186/s12885-016-2503-y)
Supplement: Additional file 2: Table S2. — Clinical and histopathological characteristics of the vSCC patients related to the course of the disease. (DOCX 13 kb) [file 12885_2016_2503_MOESM2_ESM.docx]

Supplementary Table 2. Clinical and histopathological characteristics of the vSCC patients related to the course of the disease.

| *Clinical and histopathological features* | | *No recurrence*  n= 69 (81.2%) | *Local recurrence*  n=13 (15.3%) | *Groin recurrence*  n=3 (3.5%) |
| --- | --- | --- | --- | --- |
| *Age, years, median (range)* | | 68 (36-85) | 73 (52-82) | 78 (63-85) |
| *Depth of invasion, mm,*  *median (range)* | | 7 (0.5-16) | 7,75 (2-18) | 9 (6-10) |
| Grade G1 | | 24 | 3 | 1 |
| Grade G2 | | 31 | 6 | 0 |
| Grade G3 | | 14 | 4 | 2 |
| FIGO 2009 | Ia | 1 | 0 | 0 |
|  | Ib | 39 | 3 | 1 |
|  | II | 1 | 1 | 0 |
|  | IIIa | 7 | 0 | 1 |
|  | IIIb | 14 | 6 | 0 |
|  | IIIc | 5 | 1 | 1 |
|  | IVa | 2 | 2 | 0 |
