# Supplementary material for: The overexpression of p16 is not a surrogate marker for high-risk human papilloma virus genotypes and predicts clinical outcomes for vulvar cancer
Source: BMC Cancer. 2016 Jul 13;16:465. doi: 10.1186/s12885-016-2503-y (PMC4944532; doi:10.1186/s12885-016-2503-y)
Supplement: Additional file 3: Table S3. — HPV genotype distribution and p16 status in 85 vSCC cases. This file contains the dataset supporting the conclusions. (DOCX 13 kb) [file 12885_2016_2503_MOESM3_ESM.docx]

Supplementary Table 3. HPV genotype distribution and p16 status in 85 vSCC cases

(All samples were sufficient for HPV DNA testing).

| sample | Age, years | HPV subtype | P16 status | sample | Age, years | HPV subtype | P16 status |
| --- | --- | --- | --- | --- | --- | --- | --- |
| 01/85VC | 67 | 56 | p16+ | 44/85VC | 68 | negative | p16+ |
| 02/85VC | 60 | 33 | p16+ | 45/85VC | 85 | 16 | p16+ |
| 03/85VC | 73 | negative | p16- | 46/85VC | 62 | negative | p16- |
| 04/85VC | 37 | negative | p16- | 47/85VC | 67 | 16, 18 | p16- |
| 05/85VC | 63 | negative | p16- | 48/85VC | 66 | 16 | p16- |
| 06/85VC | 69 | negative | p16- | 49/85VC | 68 | negative | p16- |
| 07/85VC | 55 | negative | p16- | 50/85VC | 47 | negative | p16- |
| 08/85VC | 47 | 16 | p16- | 51/85VC | 77 | negative | p16+ |
| 09/85VC | 70 | negative | p16- | 52/85VC | 73 | 16 | p16- |
| 10/85VC | 74 | negative | p16- | 53/85VC | 73 | negative | p16+ |
| 11/85VC | 57 | 16 | p16+ | 54/85VC | 73 | 16 | p16+ |
| 12/85VC | 44 | negative | p16+ | 55/85VC | 55 | 16 | p16- |
| 13/85VC | 76 | negative | p16- | 56/85VC | 79 | 16 | p16- |
| 14/85VC | 65 | negative | p16+ | 57/85VC | 82 | 16 | p16- |
| 15/85VC | 67 | negative | p16- | 58/85VC | 71 | 16 | p16+ |
| 16/85VC | 63 | 6 | p16- | 59/85VC | 77 | 16 | p16+ |
| 17/85VC | 77 | 39, 51 | p16+ | 60/85VC | 50 | 16 | p16- |
| 18/85VC | 80 | negative | p16- | 61/85VC | 61 | negative | p16- |
| 19/85VC | 71 | negative | p16- | 62/85VC | 82 | 16 | p16+ |
| 20/85VC | 78 | negative | p16- | 63/85VC | 85 | 16 | p16+ |
| 21/85VC | 76 | negative | p16- | 64/85VC | 70 | 16 | p16- |
| 22/85VC | 82 | negative | p16- | 65/85VC | 36 | negative | p16+ |
| 23/85VC | 79 | 16 | p16- | 66/85VC | 48 | 16 | p16+ |
| 24/85VC | 63 | negative | p16- | 67/85VC | 64 | negative | p16- |
| 25/85VC | 58 | negative | p16- | 68/85VC | 71 | negative | p16- |
| 26/85VC | 66 | 16 | p16+ | 69/85VC | 72 | 33 | p16- |
| 27/85VC | 70 | negative | p16+ | 70/85VC | 79 | negative | p16+ |
| 28/85VC | 59 | negative | p16+ | 71/85VC | 85 | 16 | p16+ |
| 29/85VC | 67 | 16 | p16+ | 72/85VC | 40 | 16 | p16- |
| 30/85VC | 77 | negative | p16- | 73/85VC | 70 | 16 | p16+ |
| 31/85VC | 70 | negative | p16- | 74/85VC | 58 | 16 | p16+ |
| 32/85VC | 55 | 16 | p16+ | 75/85VC | 80 | negative | p16- |
| 33/85VC | 71 | negative | p16- | 76/85VC | 50 | negative | p16+ |
| 34/85VC | 61 | 16 | p16- | 77/85VC | 60 | 16 | p16+ |
| 35/85VC | 68 | negative | p16+ | 78/85VC | 51 | negative | p16+ |
| 36/85VC | 75 | 16 | p16+ | 79/85VC | 52 | 16 | p16+ |
| 37/85VC | 57 | negative | p16+ | 80/85VC | 65 | 16 | p16- |
| 38/85VC | 76 | negative | p16- | 81/85VC | 68 | negative | p16- |
| 39/85VC | 56 | 16 | p16- | 82/85VC | 82 | negative | p16- |
| 40/85VC | 80 | 33 | p16+ | 83/85VC | 68 | negative | p16- |
| 41/85VC | 77 | negative | p16- | 84/85VC | 53 | negative | p16+ |
| 42/85VC | 47 | negative | p16- | 85/85VC | 81 | 16 | p16+ |
| 43/85VC | 75 | negative | p16- |  | | | |
